# Amalgam tattoo versus melanocytic neoplasm - Differential diagnosis of dark pigmented oral mucosa lesions using infrared spectroscopy

**DOI:** 10.1371/journal.pone.0207026

**Published:** 2018-11-06

**Authors:** Johannes Laimer, Raphael Henn, Tom Helten, Susanne Sprung, Bettina Zelger, Bernhard Zelger, René Steiner, Dagmar Schnabl, Vincent Offermanns, Emanuel Bruckmoser, Christian W. Huck

**Affiliations:** 1 University Hospital for Craniomaxillofacial and Oral Surgery, Innsbruck, Austria; 2 Institute of Analytical Chemistry and Radiochemistry, Innsbruck, Austria; 3 Institute of Pathology, Medical University, Innsbruck, Austria; 4 University Hospital for Dermatology, Venereology and Allergology, Innsbruck, Austria; 5 University Hospital for Dental Prosthetics and Restorative Dentistry, Innsbruck, Austria; 6 Independent researcher, Salzburg, Austria; Drexel University, UNITED STATES

## Abstract

**Background:**

Dark pigmented lesions of the oral mucosa can represent a major diagnostic challenge. A biopsy is usually required to determine the nature of such intraoral discolorations. This study investigates the potential use of infrared spectroscopy for differential diagnosis of amalgam tattoos versus benign or malignant melanocytic neoplasms.

**Materials and methods:**

For this retrospective study, formalin-fixed paraffin-embedded tissue (FFPE) specimens of dark pigmented lesions concerning the oral mucosa or the lip were investigated using mid infrared spectroscopy. The samples were chosen from patients who had undergone a mucosal biopsy at the University Hospital Innsbruck (Austria) between the years 2000 and 2017. Principal component analysis was used for data exploration. Evaluation was based on the superimposition of the recorded spectra and the corresponding histologic slides.

**Results:**

In total, 22 FFPE specimens were analyzed. Clear differences were found between amalgam and non-amalgam samples. A general weakening of the penetrating infrared radiation allowed for unspecific discrimination between these two classes. An overall accuracy in predicting the correct class of 95.24% was achieved.

**Conclusion:**

Infrared spectroscopy appears to be a suitable technique to differentiate between amalgam tattoos and melanocytic lesions in FFPE samples. It could potentially be applied in vivo, too, serving as a non-invasive diagnostic tool for intraoral dark pigmented lesions.

## Introduction

Pigmented lesions of the oral mucosa are a common finding[1]. Exogenous factors like amalgam tattoos represent a major part of these lesions[1]. Most amalgam tattoos are due to dental treatments when metal particles accidentally deposit in open oral wounds or are dashed like a shrapnel into the oral mucosa during tooth preparation procedures[2]. Further, a galvanic element can form if various metals are used as dental restorative material[3]. Thereby, the ignoble metal dissolves and precipitates as an inorganic salt in the oral mucosa. Due to the lymphatic transport of metallic material, the discoloration can increase in size giving the impression of a growing malignant neoplasm[4]. Only in presence of a clear medical/dental history, a dark pigmented lesion can be assumed to be no more than a harmless amalgam tattoo. Bearing in mind that any dark pigmented lesion could not only be a benign discoloration but could potentially represent a melanoma[4–6], a mucosal biopsy for definitive histological diagnosis is usually required[5, 7, 8].

Regarding the differential diagnosis of dark pigmented intraoral lesions, a great variety of etiologies exist. Firstly, exogenous factors can cause discoloration due to local injuries or deposit of amalgam material. Also, systemically induced pigmentation following drug intake or poisoning (e.g. argyrosis) can be found.

Secondly, endogenous factors associated with increased melanin production can be seen in dark-skinned people[9], and are also typically found in the context of certain syndromes such as Addison's disease, Albright syndrome, Xeroderma pigmentosum, or Peutz Jeghers syndrome[10].

The third group of dark pigmented intraoral lesions is represented by melanocytic benign (e.g. nevus) or malignant (melanoma) neoplasms[11]. Details regarding different etiologies and clinical features are depicted in the following paragraphs.

In order to provide the necessary clinical background of this study, amalgam tattoos and melanocytic neoplasms will be presented in some detail in the following paragraphs.

### Amalgam tattoos

Although its importance has been on the decline for many years, amalgam is still used as a filling material in dentistry. Non-gamma-2 amalgam represents the standard type of this compound material today due to its higher chemical stability and resistance regarding corrosion compared to the formerly used copper amalgam. The compounds include silver (≥ 40%), tin (≤ 32%), copper (≤ 30%), indium (≤ 5%), mercury (≤ 3%), and zinc (≤ 2%)[12].

For several reasons, amalgam is still in use as it has some uncontestable advantages over other filling materials (in particular composites). It is easy to use, less sensitive to moist conditions in processing, very durable and cheaper than composite material. Disadvantages of amalgam include the esthetically unfavorable color and the need for retentive preparation which may require removal of additional tooth substance that could be preserved when using composites.

The main reason why this filling material has been discredited for decades by the public is the fear of significant mercury release into the body, although there is no scientific evidence to date proving a harmful effect of any component[13–15].

In the process of inserting or removing amalgam fillings, during extraction of teeth containing amalgam fillings, or during tooth preparation, metallic particles can accidentally be deposited in the oral mucosa and appear as dark pigmented lesions.

The constellation of a noble metal (e.g. gold) in close proximity to an amalgam restoration may function as a galvanic element, provided that electric charges are exchanged between both materials. This significantly speeds up the corrosion process of the less noble metal[16]. In particular, tin undergoes increased oxidation when exposed to galvanic current[3, 17, 18]. Apart from tin, oxidation also concerns other metallic components like copper, silver, and mercury[17]. Released ions can precipitate in the surrounding soft tissue and appear as dark discoloration. In many instances, these deposits are so fine that they are not visible radiologically.

### Benign melanocytic lesions

Melanotic macules represent the most common intraoral benign dark pigmentation[19] and are due to melanin deposits without increase in the number of melanocytes[20, 21]. On average, they measure around 7 millimeters in diameter[22]. They usually occur as a single lesion affecting the palate, the vestibular mucosae, the gums, and the lips.

Melanotic nevi are benign lesions composed of cells derived from the neural crest. The etiopathogenesis of these intraoral lesions is poorly understood. Melanocytic or—when nested—also nevus cells can be found in the skin as well as in mucous membranes (including the intraoral mucosa). In contrast to melanocytic nevi of the skin, intraoral lesions are relatively rare. There are various different types of nevi including junctional, compound, combined, congenital, blue, cellular blue, Spitz nevus and many other variants more[23].

### Malignant melanocytic tumor (melanoma)

Melanoma develops following malignant transformation of melanocytes[24]. In contrast to cutaneous melanomas, exposure to sunlight does not play a significant role etiologically. The molecular mechanisms involved in the development of oral mucosal melanomas are poorly understood[25]. Epidemiologically, it is the deadliest form of primary skin cancer. The typical age is from 50 years onward, and the highest incidence is found in the Japanese population[26].

Melanomas occurring in mucosal sites (including the oral cavity) are particularly aggressive and have an even poorer prognosis. Maxillary gingiva and hard palate represent the most common localization of intraoral melanoma, and its clinical features vary considerably. It can appear as plaque, macule, or mass, either irregular or well-circumscribed, and diffusely or focally pigmented. Amelanotic forms completely lack pigment[27, 28]. Multifocal pigmentation is also possible and appears in the presence of amelanotic and melanotic areas within one lesion.

Although some patients may remain asymptomatic for a long period of time, possible general oncologic signs and symptoms comprise tooth mobility, root resorption, bone loss, anesthesia or paresthesia, pain and ulceration[24]. Considering this great variety of clinical features, and despite the fact that intraoral melanoma accounts for less than 1% of all melanomas[29] and for about 10% of head and neck melanomas only[24], a biopsy is usually indicated to prove or rule out this malignancy (especially in case of a persistent, solitary, focally pigmented intraoral lesion). Once the diagnosis of a melanoma has been established, it is to be determined whether the lesion is of primary or secondary origin. Surgical resection still remains the treatment of choice[25, 30] and is sometimes combined with adjuvant radiation and chemotherapy. The 5-year survival rate ranges from 15 to 40%[25, 26, 29].

### Infrared spectroscopy

Infrared (IR) spectroscopy is a modern analytical technique enabling molecular imaging of complex samples. This method is based on the absorption of infrared radiation by vibrational transitions in covalent bonds[31]. It allows for the acquisition of high-resolution images of the in-situ distribution of various molecules including carbohydrates, proteins, lipids, cholesterols, phospholipids, nucleic acids and small molecules. Time-consuming extraction, purification and separation steps are not required, and the topographic integrity of the tissue can be preserved[32]. Qualitative and quantitative information from heterogeneous samples can be gained as the individual infrared spectrum of any compound represents a unique fingerprint-like molecular pattern[33, 34].

The use of array detectors enables efficient scanning of complex samples and constructing a hyperspectral cube where lateral as well as spectral information is contained. Coupling of this data to sophisticated analysis tools enables new insights into the chemical composition of the respective samples. Infrared imaging is a growing field of research and has its applications in biomedical, material analytics and many more.

The basic principle of IR imaging spectroscopy is the combination of a microscope to an IR spectrometer[35]. During measurement, diffraction, refraction, reflection and absorption effects play a more dominant role than in its macroscopic counterpart. Microscopes nearly consist of the same parts with regard to their optical analogues. From a spectral point of view, several components are required including a light source (single polychromatic thermal source), a splitter (Fourier transform, tunable filter or diffraction grating), and a detector (uncooled InGaAs or cooled mercury cadmium telluride). Microspatial imaging of highly complex samples, high sensitivity, high selectivity, fast data acquisition, simple sample preparation and analysis as well as fully automated examination and computer enhanced visualization represent the main assets of this technique. Extrapolating this knowledge to fiber optic probe measurements is highly suitable for further development towards a fast analytical tool enabling analyses within only a few seconds.

### Study purpose

The aim of this study was to investigate paraffin-embedded specimens of dark pigmented oral mucosa lesions by mid infrared (MIR) spectroscopy to evaluate the potential use of this technique in distinguishing metallic deposits from melanocytic lesions. Although this is only a preclinical proof-of-principle study, it can be assumed that this technique may be applied in vivo, too, using flexible probes. This would provide a non-invasive technique potentially avoiding the need for an intraoral biopsy.

## Materials and methods

### Samples

22 formalin-fixed paraffin-embedded (FFPE) specimens of dark pigmented lesions concerning the oral mucosa or the lip were investigated in this retrospective study. The diagnoses comprised amalgam tattoo, benign and malignant melanocytic neoplasms. The paraffin-embedded specimens were chosen from patients who had undergone a mucosal biopsy at the University Hospital Innsbruck (Austria) between the years 2000 and 2017. Histological reports providing the final diagnosis of the lesions were retrieved. Hereby we confirm that the responsible ethics committee in charge (i.e., Ethics Committee of the Medical University Innsbruck, Austria) specifically approved this study (reference number, 1128/2017). It has been conducted according to the principles expressed in the Declaration of Helsinki. Additionally, an informed consent form regarding the use of the respective specimens for scientific purpose had been signed by all patients. From each specimen, a 4 μm thick slice was obtained (using a microtome) and mounted onto a CaF_2_ sample carrier being transparent to MIR radiation. In addition, a Hematoxylin and Eosin (HE) staining procedure was carried out for all specimens, and the respective slides were digitalized using a Pannoramic 3DHISTECH scanner. The areas corresponding to amalgam tattoos, benign or malignant melanocytic neoplasms were digitally marked by an experienced pathologist. Samples for spectroscopy measurements were not stained.

### Mid infrared spectroscopy (MIR) measurements

Prior to data acquisition, a chemical deparaffinization step was performed using octane with a purity of ≥ 98.0% (Sigma Aldrich, Buchs, Switzerland) for 4 hours at a temperature of 40°C and dried afterwards as described by Pallua et al [36]. All MIR images were recorded in transmission mode using a commercially available infrared microscope Spectrum Spotlight 400 (Perkin-Elmer, Massachusetts, USA). Spectral data were recorded using the software “Spectrum IMAGE R1.8.0.0410” with a spectral resolution of 4 cm^-1^ and a pixel size of 25 μm. Before each measurement, a background spectrum was recorded outside the sample area to calculate a ratio[36]. Each final spectrum consisted of two co-added scans. The result of the MIR imaging measurement was a hyperspectral cube composed of both a local and a spectral information. The measurement time per sample strongly depends on the size of the sample and the used pixel size on the instrument. The average time for scanning one slide was 30 minutes. Each recorded pixel therefore had an associated MIR spectrum representing the local chemical information. By superimposing the previously gained information from the HE stained histologic slide with the recorded spectra, the development of a multivariate discrimination model could be achieved.

### Data analysis

Principal component analysis (PCA) was used as an unsupervised data exploration method to gain insights into the data. This method was first described by Karl Pearson in 1901 and widely applied since then[37]. In the year 1960, it found its way into chemistry and was shown to be useful for a wide variety of chemical datasets including spectroscopy[38]. Nowadays, many commercial and free software packages are available to perform PCA and can be found in the web. For the present work, the software package The Unscrambler X V10.5 (64bit) (CAMO Software, Oslo, Norway) was used for all multivariate data analyses shown.

PCA is a bilinear modeling method that provides an interpretable overview of the main information contained in a multidimensional table. It is also known as a projection method because it takes information carried by the original variables and projects them onto a smaller number of latent variables called Principal Components (PC). Each PC explains a certain amount of the total information contained in the original data, and the first PC contains the greatest source of information in the data set. Each subsequent PC contains, in order, less information than the previous one. By plotting PCs, important sample and variable interrelationships can be revealed leading to the interpretation of certain sample groupings, similarities or differences (The Unscrambler X 10.5. Introduction to Principal Component Analysis (PCA), CAMO Software AS, Oslo, Norway). The interested reader is redirected to relevant literature to gain insights about the theoretical background and basic principles of PCA[39].

LDA is the simplest of all possible classification methods that are based on Bayes’ formula. From Bayes’ rule, one develops a classification model assuming that the probability distribution within all groups is known, and that the prior probabilities for groups are given, and sum to 100% over all groups. It is based on the normal distribution assumption and the assumption that the covariance matrices of the two (or more) groups are identical. This means that the variability within each group has the same structure. The only difference between groups is that they have different centers. LDA considers both within-group variance and between-group variance. The estimated covariance matrix for LDA is obtained by pooling covariance matrices across groups. When the variability of each group does not have the same structure (unequal covariance matrix), the shape of the curve separating groups is not linear, and therefore quadratic discriminant analysis will provide a better classification model. The distance of observations from the center of the groups can also be measured using the Mahalanobis distance (The Unscrambler X 10.5. Introduction to Principal Component Analysis (PCA), CAMO Software AS, Oslo, Norway). Again, the theoretic details regarding this method are documented elsewhere and skipped at this point. The interested reader is redirected to existing literature[40]. The available samples were split into a calibration (2/3 of total samples) and a validation set (1/3 of total samples).

## Results

Clinical details regarding the histologically confirmed number of amalgam tattoos as well as the number of both benign and malignant melanocytic lesions can be found in Table 1.

**Table 1:** Total number of amalgam tattoos, benign and malignant melanocytic lesions of all 22 cases.

Case number	Gender (F: female, M: male)	Age (in years)	Location	Amalgam	Benign melanocytic lesions	Malignant melanocytic lesions
1	F	39	lower lip		X	
2	F	88	cheek			X
3	F	52	lower lip		X	
4	F	32	upper lip		X	
5	M	53	lower lip		X	
6	M	77	lower lip		X	
7	F	77	lower lip		X	
8	F	65	upper lip		X	
9	M	26	lower lip		X	
10	M	38	lower lip		X	
11	F	80	oral cavity		X	
12	M	51	cheek	X		
13	F	79	cheek	X		
14	F	77	mandible	X		
15	F	63	mandible	X		
16	M	82	maxilla	X		
17	F	57	oral cavity	X		
18	M	78	oral cavity	X		
19	F	68	oral cavity	X		
20	M	56	oral cavity	X		
21	F	36	cheek	X		
22	M	49	maxilla	X		

**Fig 1:**
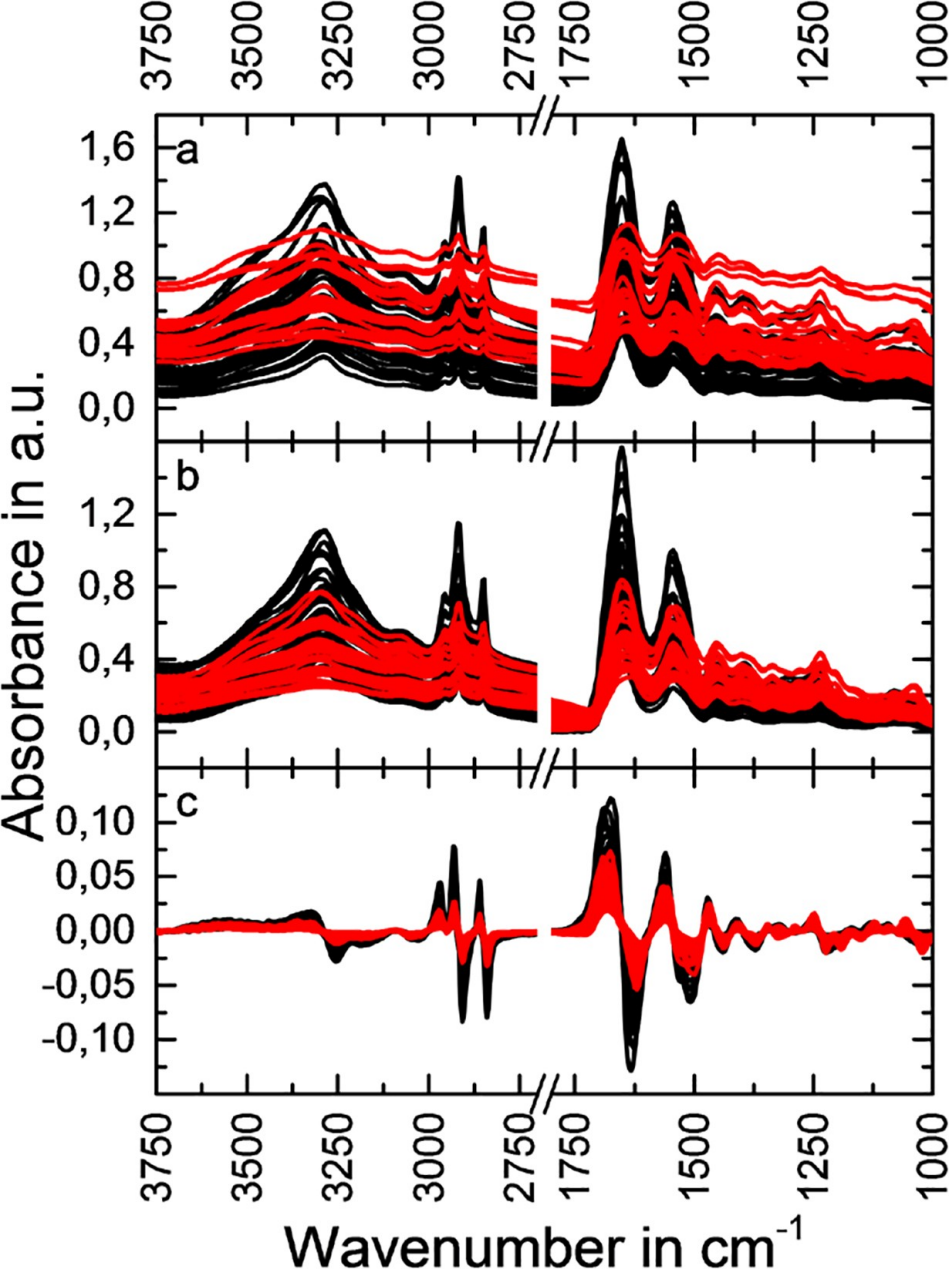
MIR spectra. Raw experimental MIR spectra (absorbance in arbitrary units (a.u.) versus wavenumbers) of the investigated samples are shown (a). Each spectrum represents the average of one region of interest (ROI). The color coding represents the assignment to one of the two investigated classes which are amalgam (red) or non-amalgam (black). The spectra after baseline correction (b) and, additionally, the first Savitzky Golay derivative (c) are shown.

Experimental raw (Fig 1A) and pre-processed (Fig 1B and 1C) MIR spectra are shown. Each of these spectra represent a region of interest (ROI) by averaging all spectra within this region. Three ROIs per sample were defined. These regions were selected by taking the HE stained tissue sections into account. ROIs were solely defined by the presence of pigmentation. To simplify the overall workflow, structural differences were not considered. The selected features for analysis correspond to the described molecular vibrations of Fig 1

As expected, the major peaks corresponded to Amide I, Amide II and Amide III bands of proteins. Furthermore, C-H as well as P-O vibrations could be observed. A detailed spectral band assignment is depicted in Table 2 as described by Pezzei et al [41]. Clear differences can be seen between the two defined classes amalgam (A) and non-amalgam (nA) after the pre-processing steps (Fig 1C). Baseline correction and the first derivative via Savitzky Golay (7 smoothing points 2^nd^ order polynomial)[42] were used for the present application. As apparent from Fig 1, the differences between the two classes were not defined by distinguishable molecular vibrations but by the overall intensity of the tissue vibrations. This can be explained by the inorganic nature of the incorporation itself. A general weakening of the penetrating infrared radiation was observed allowing for unspecific discrimination between the two classes. Besides, there were no observable differences between the benign and malignant melanocytic lesions.

**Table 2:** Major spectral peaks of tissue samples.

Wavenumber in cm^-1^	Assignation
≈ 3300	Amide A, *ν*_*N*−*H*_ of proteins
≈ 3100	Amide B, *ν*_*N*−*H*_ with 1. overtone of the amide I band resonant (Fermi), proteins
≈ 3010	*ν*_*C*−*H*_ (lipids), cholesterols, esters
≈ 2920 and 2850	*ν*_*C*−*H*_ (> CH_2_, methyl) antisymmetric/symmetric, lipids, proteins, carbohydrates, nucleic acids
≈ 2956 and 2872	*ν*_*C*−*H*_ (CH_3_, methyl) antisymmetric/symmetric, lipids, proteins, carbohydrates, nucleic acids
≈ 1745–1735	*ν*_*C* = *O*_, esters and phospholipids
≈ 1620–1695	Amide I-band, proteins
≈ 1550	Amide II-band, proteins
≈ 1400	*ν*_*C* = *O*_ of COO^-^ groups, fatty acids and amino acids
≈ 1360–1260	Amide III-band (mainly C-N stretching) with contributions from $\nu_{CH_{2}}$ of carbohydrate residues
≈ 1350–1260	$\nu_{PO^{2-}}$
≈ 1310–1240	Amide III-band, proteins
≈ 1250–1220	*ν*_*P* = *O*_ symmetric of the PO^2−^ groups, phospholipids, nucleic acids
≈ 1225	$\nu_{PO^{2-}}$ antisymmetric of nucleic acids and phospholipids
≈ 1185–1120	C-O ring vibration of nucleic acid “sugars”
≈ 1084	*ν*_*P* = *O*_ symmetric of the PO^2-^ groups nucleic acids, phospholipids

**Fig 2:**
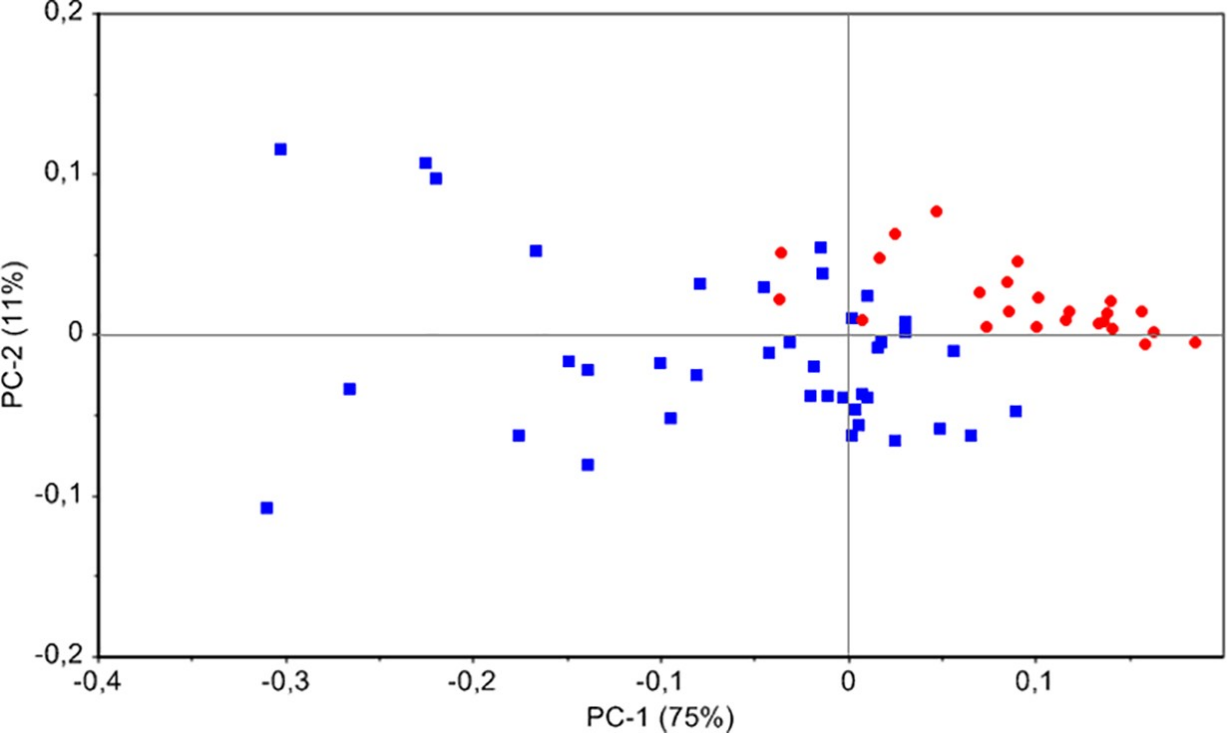
PCA scores plot of ROIs explaining 86% of the total variance in the data. Amalgam (red) and non-amalgam (blue) regions can be discriminated visually with few exceptions.

Based upon the observed differences, a pattern recognition method can be applied to evaluate the performance of MIR spectroscopy regarding the present problem. The scores plot from the PCA provided information regarding all analyzed samples and revealed dissimilarities (Fig 2).

Besides the scores plot, the loadings plot (S1 Fig) should be analyzed to see which of the submitted variables are of greatest importance, allowing direct interpretation of molecular vibrational contributions to the trend seen in the scores plot. Looking at the loadings, it can be seen that almost all variables for molecular vibrations strongly contributed to the pattern shown in Fig 2. This supports the finding (already mentioned earlier) that no distinct features contributed to the separation but the overall weakening of the transmitted infrared light. As apparent from Fig 2, the two classes can be separated. Nevertheless, there are three samples situated in the blue cluster. One possible explanation is that the actual amalgam incorporation is too small in relation to the total area and therefore has negligible effect on the average spectrum. This would lead to a positioning in the blue (nA) region. However, PCA is not a classification method but a data exploration tool. Therefore, an appropriate method must be employed to assess prediction accuracy of the herein presented method. Since each spectrum represents an individual locally different ROI, independence of each spectrum was assumed. This justifies the neglection of the fact that one tissue section is represented by three spectra. If this was not true one would have to consider the similarity between three ROIs among one sample by predicting the three ROIs per section simultaneously without their presence in the calibration. The LDA yielded 95.24% accuracy in the calibration process using quadratic function and four components. The test-set prediction matched one of the 21 test samples wrong. This resulted in an overall accuracy in predicting the correct class of 95.24%.

**Fig 3:**
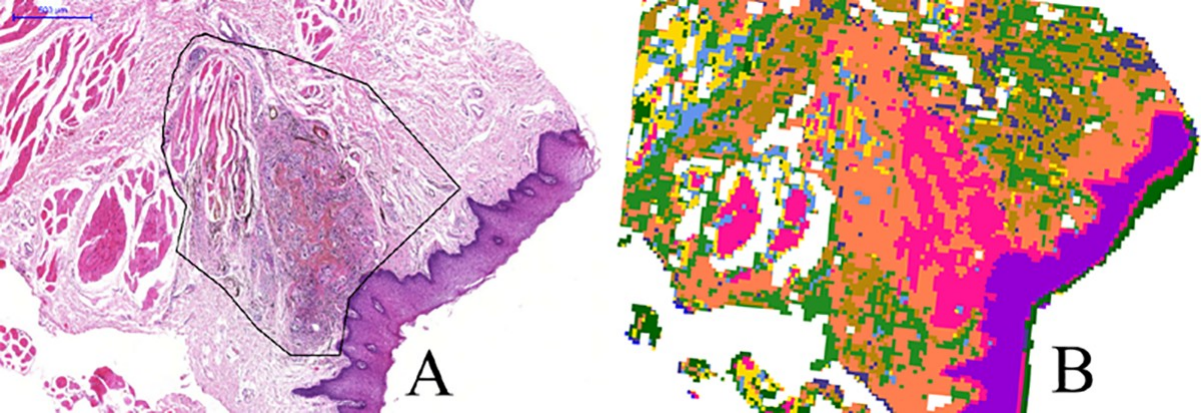
**Correlation between the HE stained tissue section (3a) and the corresponding false color infrared image after hierarchical cluster analysis using 10 clusters (3b).** The amalgam containing area is framed in the HE stained section.

Additionally, a visual correlation between the infrared imaging result and the classically HE stained tissue section is possible. Therefore, multivariate clustering methods were employed. The result of this procedure is shown in Fig 3. Comparing the false color image (Fig 3B) with the stained tissue section (Fig 3A), histological features can be discriminated using infrared spectroscopy in combination with clustering. Pre-treatments before multivariate cluster analysis (hierarchical cluster analysis—HCA) were conversion to absorbance spectra followed by vector normalization and second order Savitzky-Golay derivative (second order polynomial, 13 smoothing points).

## Discussion

The goal of this study was to evaluate the use of FTIR (Fourier Transform Infrared) spectroscopy to distinguish between intraoral amalgam tattoos (intramucosal metallic foreign bodies) and melanocytic lesions of the oral mucosa.

Dark pigmented intraoral lesions are commonly found and, most of the time, represent harmless amalgam tattoos. However, there is a multitude of other etiologies the differential diagnosis of which can be challenging[7, 43]. To date, intraoral biopsy and subsequent histopathologic examination still represent the diagnostic gold standard.

Recent literature has demonstrated FTIR spectroscopy to be a potential diagnostic tool in various medical fields. The advantages of this rapidly evolving technique include a high resolution near cellular level, the capacity of determining the biodistribution of proteins, peptides, lipids, and carbohydrates not requiring any pretreatment, and the possibility to gain molecular structure information[44].

In a clinical study investigating papillary thyroid carcinoma and goiter tissue, FTIR spectroscopy and canonical discriminant analysis was combined for diagnostic purpose. Using this combination, a fast and accurate intraoperative discrimination of malignant tissue versus benign thyroid nodules could be shown. Sensitivity, specificity, and accuracy of the discriminants were reported to be 83.3%, 95.2%, and 91.67%, respectively[45].

Comparable findings were reported in prostate cancer where benign and malignant areas could be distinguished with a resolution of 6.25 μm × 6.25 μm in frozen sections[41]. Similarly, surgical specimens of oral squamous cell carcinoma tissue can be characterized using FTIR imaging[46]. In yet another study, it could be demonstrated that referring to a single infrared spectroscopic image from a lymph node biopsy, cells can be distinguished to a similar level as compared to immunohistochemical stains and manual recognition using optical microscopy[47]. Evaluating the potential of FTIR spectroscopy in early diagnosis of skin cancer (including melanoma, trichoblastic carcinoma/basal cell carcinoma, and melanocytic nevus), it was proposed that certain frequency bands could be used as "diagnostic marker" bands for DNA forms[48].

The above findings would suggest that also lesions of the intraoral mucosa may potentially be distinguished by use of FTIR. As already mentioned earlier, the focus of our study was set on the discrimination of intramucosal metallic amalgam deposits from melanocytic lesions in general.

Apart from the method used in our study, various other techniques to detect amalgam residues have been reported, one of which is in vivo intraoral reflectance confocal microscopy. This technique has been described in a case report where an amalgam tattoo was imaged using an intraoral probe attached to a handheld reflectance confocal microscopy device[49]. Since amalgam granules may not be directly visible using reflectance confocal microscopy due to their relatively deep location (200–300 μm), the tentative diagnosis is rather based on the presence of bright dots (lymphocytes) and plump cells (macrophages) indicating a foreign body reaction around the metallic deposits. Apart from this case report, there are currently no clinical studies on this novel approach.

Another technique to diagnose amalgam tattoos would be conventional radiography. However, the size of the foreign body is the main limiting factor since small metallic particles cannot be visualized radiographically[50–52].

To the best of our knowledge, the herein presented study describes the first attempt to distinguish amalgam tattoos from melanocytic lesions employing FTIR spectroscopy in FFPE tissue samples. Our results suggest that such a distinction is possible. In contrast to other studies where the diagnosis referred to the appearance of a new spectroscopic band, the herein presented technique relies on the general decrease of vibration in the analyzed areas of interest. The appearance of new bands in melanoma cells can be explained by structural remodeling processes on a molecular level[48]. In our study, such changes were not accounted for since the inorganic nature of the metallic particles was found to be a sufficiently reliable distinctive feature already.

It could be criticized that MIR may not be ideally suited for the present task. However, if further research towards in vivo experiments is considered, other options offering a similar flexibility are rare. Flexible probes enabling measurements directly on the suspicious lesion are available for NIR, MIR and Raman. Imaging itself is just the first step to evaluate the principle possibility of differentiation. Ideal techniques would yield clear elemental response without interferences.

Further, it may be criticized that spectroscopic measurements have been performed on histopathologic specimens in this preclinical study and not in vivo which is of limited or even no use for patients since the diagnosis can be reliably established assessing the respective histological slides. However, looking at relevant international literature[53, 54], it can be hypothesized that spectroscopic measurements may be performed in vivo using suitable probes in the not too far future. Besides, we mainly consider the herein presented results as preliminary basic research showing the capability of MIR spectroscopy for this task. Based on these results, the use of flexible probes in vivo can be seen as the next step.

Because 95,24% of the samples were correctly classified with the presented workflow, an easier to handle and closer to the reality experimental realization could have a promising future and should be developed. The implementation of fiber optical probes is a thinkable option to enable measurements directly on FFPE tissue sections, blocks or even directly in patients. Furthermore, other vibrational spectroscopic technologies such as near-infrared or Raman spectroscopy are imaginable alternatives and can be combined with fiber optical probes.

In conclusion, FTIR spectroscopy seems to be a potential diagnostic tool to differentiate amalgam tattoos from melanocytic lesions. This preliminary study in FFPE tissue samples should serve as the basis for further clinical studies to aim for the in vivo use of this technique in the not too far future.

## Supporting information

S1 Fig Loadings plot. All observed molecular vibrations contribute to the observed pattern in the scores plot in Fig 2. The total amount of explained variance by the first two principal components is 86%.
(TIF)
