# Supplementary figures and images for: Amalgam tattoo versus melanocytic neoplasm - Differential diagnosis of dark pigmented oral mucosa lesions using infrared spectroscopy
Source: PLoS One. 2018 Nov 6;13(11):e0207026. doi: 10.1371/journal.pone.0207026 (PMC6219804; doi:10.1371/journal.pone.0207026)

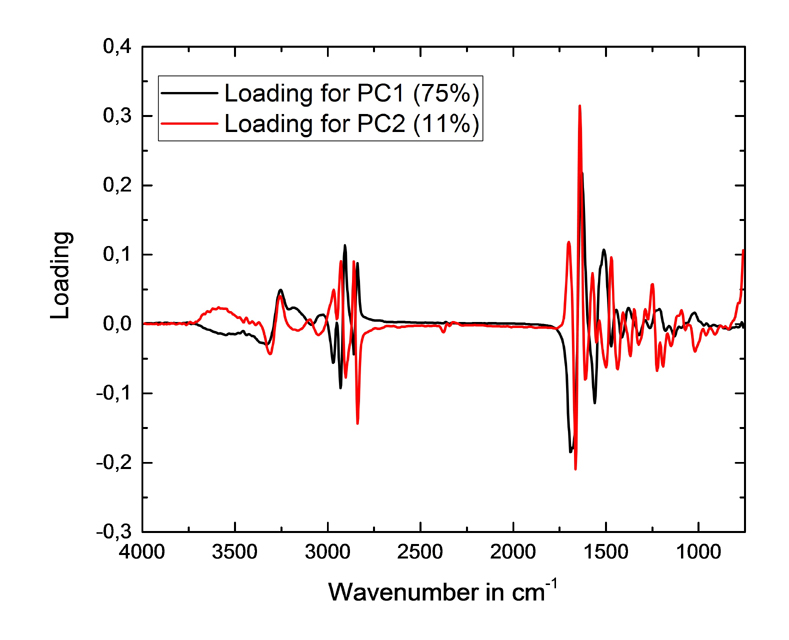

Supplement: S1 Fig — All observed molecular vibrations contribute to the observed pattern in the scores plot in Fig 2. The total amount of explained variance by the first two principal components is 86%. (TIF) [file pone.0207026.s001.tif]
